# Phylogenetic and Phylodynamic Analysis of Delta Strains Circulating in Italy

**DOI:** 10.3390/v15091791

**Published:** 2023-08-23

**Authors:** Leonidas Salichos, Claudia Minosse, Ubaldo Visco-Comandini, Chiara Taibi, Verdiana Zulian, Gianpiero D’Offizi, Nayan Pallothu, Fiona McPhee, Anna Rosa Garbuglia

**Affiliations:** 1 Biological and Chemical Sciences, New York Institute of Technology, Manhattan, NY 10023, USA; lsalicho@nyit.edu (L.S.); npalloth@nyit.edu (N.P.); 2 Virology Laboratory, National Institute for Infectious Diseases Lazzaro Spallanzani IRCCS, 00149 Rome, Italy; claudia.minosse@inmi.it (C.M.); verdiana.zulian@inmi.it (V.Z.); 3 Infectious Diseases and Hepatology Unit, National Institute for Infectious Diseases Lazzaro Spallanzani IRCCS, 00149 Rome, Italy; ubaldo.viscocomandini@inmi.it (U.V.-C.); chiara.taibi@inmi.it (C.T.); gianpiero.doffizi@inmi.it (G.D.); 4 Independent Researcher, North Devon EX31, UK; mcpheef@gmail.com

**Keywords:** hepatitis delta virus, subtypes, molecular epidemiology, phylodynamics, HDV-1

## Abstract

The hepatitis delta virus (HDV) exhibits high genetic and evolutionary variability and is classified into eight genotypes (HDV-1 to -8). HDV-1 is the most widespread genotype worldwide and includes several subtypes. It predominates mainly in Europe, the Middle East, North America, and Northern Africa, and is associated with both severe and mild forms of liver disease. In this study, we performed phylogenetic and phylodynamic analyses of HDV strains circulating in Regione Lazio, Italy, to understand when these strains were introduced into the Lazio region and to define their genetic variability in Italy. Fifty HDV RNA positive patient samples were amplified using a nested RT-PCR approach targeting the HDV R0 region and sequenced. A phylogenetic tree of patient-derived sequences and reference sequences representing HDV-1 to -8 was constructed using the GTRGAMMA model in RAxML v8. The results indicated that HDV-1 was the predominant genotype with HDV-1d being the most frequently inferred subtype. HDV-1 sequences clustering with subtypes 1b and 1e were also identified. A phylodynamic analysis of HDV-1 sequences employing a Bayesian birth-death model inferred a clock rate of 3.04 × 10^−4^ substitutions per site per million years, with a 95% Highest Posterior Density (HPD) interval of 3.45 × 10^−5^ to 5.72 × 10^−4^. A Bayesian birth-death analysis with tree calibration based on a sample dating approach indicated multiple original sources of infection (from the late 1950s to late 1980s). Overall, these results suggest that HDV sequences from the native Italian and non-Italian patients analyzed in this study represent multiple lineages introduced across a wide period. A common ancestral origin should be excluded.

## 1. Introduction

The hepatitis delta virus (HDV) is a member of the *Kolmioviridae* family and is the only human virus in the Deltavirus genus [1,2]. It is an RNA-defective virus that relies on coexisting with the hepatitis B virus (HBV) to become infectious, since the encapsulation of the HDV virion with hepatitis B surface antigen (HBsAg) envelope proteins enables viral entry into hepatocytes [3]. HDV exhibits several peculiarities, including how it is the smallest virus of all known mammalian viruses with a genome between 1672 and 1697 nucleotides (nt) in length [4]. Moreover, it is the only known human pathogenic satellite virus that does not encode for its own envelope [3,5]. The HDV genome is a single-stranded closed circular negative-sense RNA, which exists in an unbranched quasi double-stranded conformation. Each HDV genome has multiple copies of the hepatitis delta antigen (HDAg) bound to it, forming a ribonucleoprotein (RNP) complex [6]. HDAg, the only protein encoded by HDV RNA, exists in two isoforms. One is small (S-HDAg) and the other is large (L-HDAg). These proteins are essential for the initiation of viral genome replication (S-HDAg) and viral assembly (L-HDAg) [5,7].

HDV exhibits high genetic and evolutionary variability and has been classified into eight genotypes (HDV-1 to -8), with genomic differences of up to 49% [8,9]. HDV genotypes can be further split into two or more subtypes that differ by ≤10% of their sequences (≤16% for HDV-1) [10], based on the HDV R0 region. To date, at least nine subtypes have been identified when evaluating the full-length HDV genome [11]. HDV-1 is the most geographically widespread genotype. It is the predominant genotype in Europe, the Middle East, North America, and Northern Africa, and is associated with both severe and mild forms of liver disease [12]. Of the HDV-1 subtypes described to date, the most prevalent one is HDV-1d, especially in Europe and Asia, whereas HDV-1a and HDV-1b have only been detected in the Sub-Saharan geographical region. A relatively new subtype, HDV-1e, was recently identified in sequences from France [11].

HDV-2 has been detected in Russia, Eastern Asia, and North Asia, including Japan and Asia, while HDV-4 has been observed in Japan and Taiwan [13,14,15]. HDV-3 is the most distantly related genotype and is exclusively found in the Amazon Basin and considered the most infectious and pathogenic of the HDV infections. HDV-5 is predominant in West Africa, whereas HDV-6, -7, and -8 have been isolated in patients from Central Africa [10,16]. However, the emigration of people from the African continent has led to the introduction of these HDV strains into Europe, as demonstrated by a study conducted in France [10].

In 2015, the World Health Organization (WHO) estimated that 257 million people (3.5% of the world’s population) were infected with HBV [17,18]. However, for HDV, different studies have suggested a range of estimations. A 2003 review estimated the prevalence of HDV coinfection among individuals with HBV infection to be around 5% (about 13 million worldwide) [19], while in two recent meta-analyses, the global HDV prevalence was estimated to be between 0.8 and 0.98% in the general population, and 13 to 14% in the HBsAg-positive population, which corresponds to around 60 million infections globally [20,21]. Chronic co-infection with HBV and HDV is considered to cause the most severe form of viral hepatitis, leading to a rapid progression of cirrhosis, higher rates of hepatocellular carcinoma, and liver-related mortality, when compared to HBV mono-infection [22,23,24]. Generally, the detection of anti-HDV antibodies (Ab) among HBsAg-positive patients has been higher in lower-income regions such as West-Central Africa [22] and the Amazon basin, however, levels of 18–20% have been reported in Eastern Europe [13,25]. In Italy, anti-HDV Ab was detected in 24.6% of HBsAg-positive individuals during the 1980s [26]. However, a decrease in HDV detection was observed after anti-HBV vaccination was introduced. In fact, anti-HDV Ab positivity was estimated to be 14% in 1997 [27] and 8.3% in 2000 [28]. Nevertheless, a continuous increase in HDV prevalence was observed in subsequent years, reaching 10% in the first two decades of this millennium [29]. Stroffolini et al. recently reported that HDV detection in native Italians (6.4%) was significantly less (*p* < 0.001) than for immigrants (26.4%) living in Italy. Moreover, the prevalence of anti-HDV Ab-positive samples was almost double in younger (<50.1 years) than older individuals (15% vs. 8.1%), suggesting that risk factors such as drug addiction could facilitate HDV transmission. In this study, we performed phylogenetic and phylodynamic analyses on HDV strains circulating in Regione Lazio, Italy, to understand/establish when they were introduced into the region and define their genetic variability.

## 2. Materials and Methods

### 2.1. Sample Collection

Serum and plasma samples were collected from chronic HDV RNA-positive patients who visited the outpatient clinic at INMI L Spallanzani IRCCS Medical Center/Hospital, Rome (Italy), for routine clinical and virological assessment. Total RNA was extracted from 400 µL of each sample using the automated system QIASYMPHONY (QIAGEN, Hilden, Germany). HDV RNA was quantified by Real-Time PCR using the Bosphore Quantitation-Detection Kit v1 (Anatolia Geneworks, Sultanbeyli, Turkey) with a limit of detection of 100 copies/mL and the 7500 TaqMan platform with a dynamic range of 10^3^ to 10^8^ copies/mL.

### 2.2. Sequencing Analysis

HDV RNA-positive samples were amplified using a nested RT-PCR approach of the HDV R0 region [30]. First-strand cDNA was produced as follows: a 10 µL solution (1 µL of random hexamer primer (50 ng/µL), 5 µL of total RNA, 1 µL of dNTP (10 mM), and 3 µL of DEPC-treated water), was incubated at 65 °C for 5 min and placed on ice for 1 min. This solution was added to a reaction mixture comprising 10× RT buffer (2 µL), 25 mM of MgCl_2_ (4 µL), 0.1 M of DTT (2 µL), 40 U/µL of RNase OUT (1 µL), and 200 U/µL of SUPERSCRIPT III RT (0.5 µL; Thermofisher, Waltham, MA, USA). The reaction mixture was incubated at 25 °C for 10 min, 50 °C for 50 min, 85 °C for 5 min, and chilled on ice. A first round amplification was set up as follows: cDNA (5 µL), 25 mM of 1480 sense primer (5′TCATCTCGAGTCTCTTGATGGTC3′), 25 mM of 1289 antisense primer (5′GAAGGAARGGCCCTCGAGAACAAGA3′), 10× buffer (Thermofisher), 2 mM of MgCl_2_, 0.25 mM of dNTP, and 2.5 TaqGold DNA polymerase (Applied Biosystems, Inc., Singapore)) in a final volume of 50 µL. The amplification conditions were as follows: 95 °C, 10 min, followed by 35 cycles of 95 °C for 30 s, 58 °C for 30 s, 72 °C for 1 min, and a final extension at 72 °C for 7 min. An aliquot (5 µL) from this amplification was used in the semi-nested PCR, which included an inner sense primer, 889 s (5′CATGCCGACCCGAAGAGGAAAG3′), and the antisense 1289 primer described above. The amplification conditions were as described for the first round. Positive products were subsequently sequenced.

### 2.3. Reference HDV Sequences

All available genomes from the HDVDB (https://hdvdb.bio.wzw.tum.de/hdvdb/, accessed on 20 April 2023) [31] were downloaded when building the reference dataset. After removing duplicates and ubiquitous sequences without genotype or date of sampling information, the reference dataset consisted of 511 sequences spanning genotypes 1 to 8 (see Supplementary Materials).

### 2.4. Genotyping and Phylogenetic Analysis

HDV genotypes were determined by the direct sequencing of the R0 region, as previously described [30]. The resultant R0 sequences (omitting primer sequences) were compared to those in the NCBI GenBank database using nucleotide BLAST (http://blast.ncbi.nlm.nih.gov/Blast.cgi, accessed on 20 April 2023) before being edited, aligned using the program BioEdit (https://bioedit.software.informer.com/7.2, accessed on 20 April 2023), analyzed, and subsequently submitted to GenBank (accession number OR348670-OR348721).

To build multiple sequence alignments based on nucleotide data, MAFFT v.7 [32] was employed with the more accurate L-INS-i option. MEGA v11 [33] was used to calculate Maximum Likelihood (ML) estimations for different evolutionary models. GTR+G+I was the best model based on likelihood.

**Figure 1:**
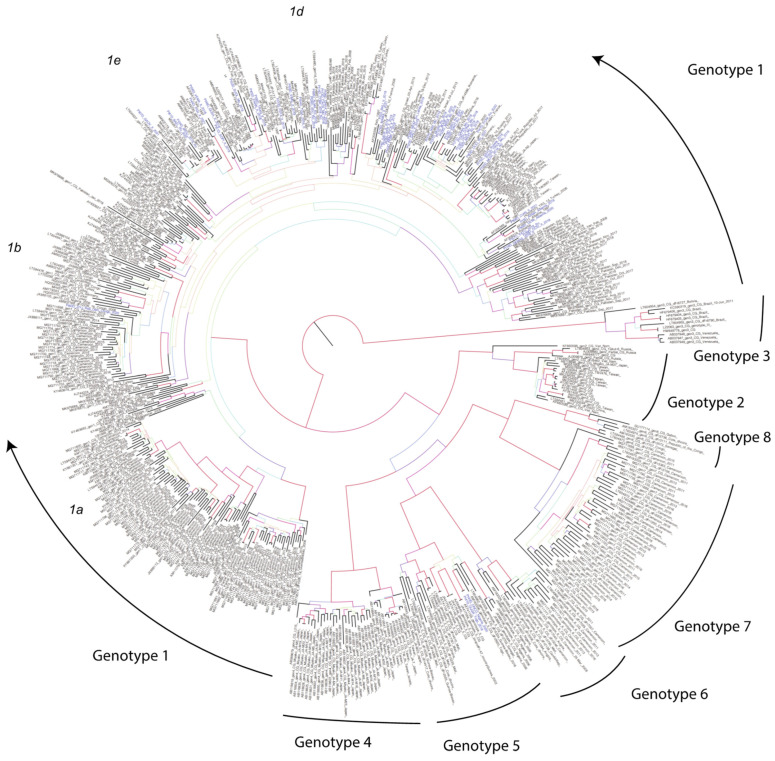
A maximum likelihood phylogenetic tree inference for 511 reference and 50 patient sequences across 8 genotypes for HDV in Italy. Reference sequence names are colored black, while Italian and non-Italian sequence names are colored blue. Branch color and width corresponds to bootstrap support, based on an HSB color spectrum [RGB_min_{204,102,102}|RGB_max_{204,102,108}]. The root has been set at midpoint. The tree was inferred with RAxML v8, and visualized with Figtree v1.4.4. Graphic illustration was implemented with Adobe Illustrator 2023. The exact color scheme corresponding to each bootstrap value is shown in Supplementary Materials.

For the ML phylogenetic analysis incorporating all genotypes, 561 sequences (50 patient sequences and 511 reference sequences) were included (see Figure 1, Supplementary Figure S1, Tables S1 and S2). A phylogenetic tree was inferred by applying 100 bootstrap replicates using the GTRGAMMA model in RAxML v8 [34].

For the phylogenetic analysis of HDV sequences with the available date of sampling information (*n* = 102; see Supplementary Materials), an ML tree was generated (Supplementary Figure S2) by applying 100 bootstrap replicates and partial deletion (90% site coverage cutoff).

### 2.5. Phylodynamic Analysis of HDV Genotype 1

**Figure 2:**
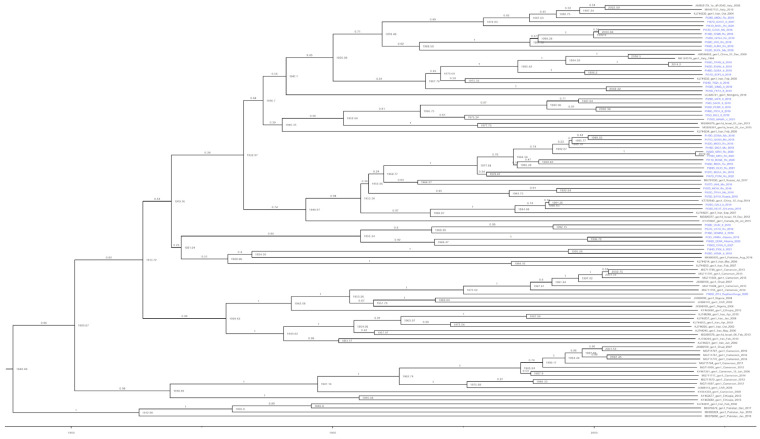
The introduction of HDV-1 in Italy. Here, we implemented a Bayesian tree inference using a birth-death population model. Dates were calibrated using date of sampling information. Branch labels show the posterior probability for each lineage. Node labels show a suggestive date based on tree calibration for each clade’s ancestral infection. Italian and non-Italian patient sequences are colored blue. The tree was inferred using BEAST v2.6.3 suite, Tracer v1.7, and TreeAnnotator. Tree calibration and visualization was implemented using Figtree v1.4.4 and sampling information.

A phylodynamic analysis was performed on the 102 HDV-1 sequences with a sampling date (see Supplementary Materials). The representation of one sequence per country was included in this analysis for a specific year (Figure 2).

**Figure 3:**
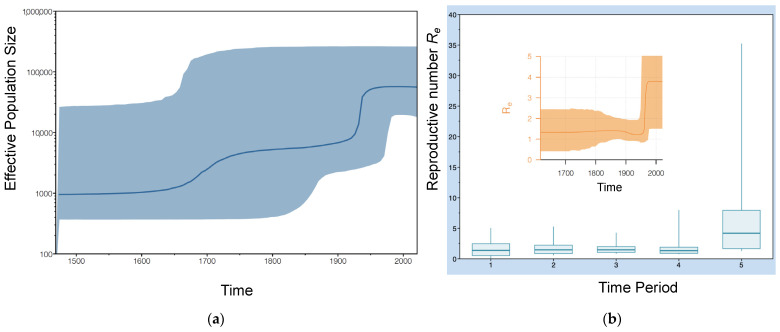
HDV phylodynamic analysis. (**a**) Estimation of the effective population size growth analysis using a Coalescent Bayesian skyline model. (**b**) Changes in effective Reproductive Number (R_e_) for HDV-1 spanning five time periods using a birth-death skyline serial model. The phylodynamic analysis was performed using BEAST v2.6.3 suite, Tracer v1.7, and BDSKY Tools package in R.

To build multiple sequence alignments based on nucleotide data, MAFFT v7 [32] was employed with the L-INS-i option. First, the Bayesian Evolutionary Analysis by Sampling Tree (BEAST) v2.6.3 [35,36] program was employed to perform a Coalescent Bayesian skyline analysis with time constraints based on the sampling dates to determine the tree prior. The strict molecular clock option and HKY+G+I site model were applied in BEAST v2.6.3 before running the Markov chain Monte Carlo algorithm for a chain length of 300 million states while sampling every 30,000. All effective sample size (ESS) values were greater than 300. It should be noted that a parallel relaxed clock analysis did not converge after 300 million states. A Bayesian skyline reconstruction was then implemented using Tracer v1.7 [37]. Based on the Bayesian skyline results, a birth-death model was selected for the tree calibration analysis (Figure 3). The strict molecular clock and BEAST Model Test site model were also selected, and the Markov chain Monte Carlo algorithm was run for 500 million states using BEAST v2.6.3 while sampling every 50,000. All ESS values were greater than 5000. Next, TreeAnnotator [38] was employed to infer the best tree with maximum clade credibility, tree type, and ancestor heights as node heights. Finally, tree management visualization and calibration were performed using Figtree v1.4.4. For estimating the reproduction rates, a birth-death skyline serial prior was applied, since data were asynchronous. Specific priors were also modified according to the following: Become Non-infectious Rate (Log Normal, S = 2) and Reproductive Number (Log Normal, S = 1.25). For the sampling proportion, a combined (natives and immigrants) number of about 5 million infectious individuals in Italy was estimated according to Caviglia et al. [39]. Therefore, the sampling Proportion prior (Beta distribution, Alpha = 1, Beta = 9999) was modified. Using Beast v2.6.3, the Markov chain Monte Carlo algorithm was run for 300 million states, while sampling every 30,000. Then, BDSKY Tools package in R [40] was employed to construct a corresponding figure (Figure 3b). All ESS values were greater than 2000.

## 3. Results

### 3.1. Phylogenetic Analysis

Demographics for the 50 sequenced HDV RNA-positive patients in Italy were analyzed. Twenty patients were native Italian and 30 patients were non-Italian. Among the non-Italians, the most prevalent ethnic groups were Romanian, Albanian, and Moldavian (Table S2). The median age was 54 years old (range 26–70 years old), and the median HDV RNA level was 1,240,875 IU/mL (range 1140->100,000,000 IU/mL).

Phylogenetic analysis of the HDV sequences from the 50 HDV RNA-positive patients and 511 reference sequences corresponding to each of the eight HDV genotypes revealed that HDV-1 predominated among the patient sequences, with HDV-1d being the most prevalent subtype (*n* = 11), although HDV-1b (*n* = 1) and HDV-1e (*n* = 3) were also detected (Figure 1).

For the 30 non-Italian patients, the majority of HDV sequences segregated into two groups. The first group included 15 closely related sequences that clustered with other HDV-1 isolates: 9 Romanian (Pt11, Pt32, Pt33, Pt35, Pt37, Pt47, Pt50, Pt63, Pt78), 5 Moldavian (Pt18, Pt19, Pt27, Pt55, Pt71), and 1 Russian (Pt75). These sequences were grouped together with reference sequences mostly from Romania and Russia. It should be noted that Pt50 and Pt78 were identical in sequence. The second group included five very closely related sequences that clustered with HDV-1d strains: four Romanian (Pt13, Pt14, Pt26, Pt68) and one Moldavian (Pt13). These sequences clustered together with a Romanian reference LT594480_dFr6801 from 2015, which interestingly did not represent an early infection for this group. An HDV-1 sequence from an Albanian patient (Pt69), as described above, was identical to a reference sequence (AF008311_Albania_1997). There were three patients with HDV sequences originating from African strains, which included two West African patients (Pt40 and Pt41) with identical HDV-5 sequences and one patient from Central Africa (Pt60) whose HDV sequence clustered with HDV-1b reference sequences.

For the 20 Italian patients, two sequences (Pt56, Pt65) clustered with AF008407_Italy_1997 from Italy). Additionally, two very closely related sequences (Pt48, Pt51), together with Pt52, also clustered within the same lineage, shown to generally contain reference sequences from Italy, Germany, Portugal, and Iran. Another two Italian sequences (Pt 59, Pt70) clustered within a lineage of sequences mostly from France and Germany. A different HDV-1d cluster included five of the non-Italian sequences (Pt13, Pt14, Pt26, Pt28, Pt68) and a subclade represented by an Italian sequence (Pt54). These sequences were closely related to LT594480_1d from Romania. Another group of four closely related Italian sequences (Pt4, Pt29, Pt43, Pt46) clustered together with reference sequences AF008383_It_1997 and AM902174_Fr_2007 from Italy and France, while a further two Italian sequences (Pt5, Pt76) were closely related to a sister group comprising one Italian (AF008386_Italy_1997) and five Turkish reference sequences, potentially representing one of the early infections for this lineage. Similarly, another lineage of closely related sequences, which included three patient sequences from Italy (Pt16, Pt39, Pt66), two from Albania (Pt3, Pt69), and one from Romania (Pt57), clustered together with Italian (AF008388_Italy_1997), Albanian (AF008311_Albania_1997), Romanian (AF008319_Ro_1997), and Turkish (HQ005372_TR3_2006) reference sequences.

Finally, three patient sequences (two Romanian, Pt34 and Pt61; one Italian, Pt67) clustered together with reference strains designated as representing HDV-1e (AM902177_Fr_2007, AM902165_Algeria_2007 and AM902179_It_2007). Other previously reported isolates in this cluster mostly included those from Italy, Spain, and France. A sister group to the HDV-1e cluster comprising two Italian sequences (Pt44, Pt58) clustered with AF008413_Italy_1997 from Italy. Similarly, two additional Italian sequences (Pt59, Pt70_) clustered in a clade of previously reported sequences mostly from France and Germany. It is unclear whether these four sequences represented HDV subtypes 1d or 1e.

The remaining patients had HDV sequences that were scattered across the phylogenetic tree clustering with different reference sequences from Western Europe. In addition, two close sequences (from Italy, Pt22, and from Sri Lanka, Pt38) clustered with a group of four Iranian reference sequences (Figure 1).

Among the Italian patient sequences, the mean genetic distance was 0.084, with a minimum value of 0.0114 and a maximum value of 0.12. Considering the sequences identified in non-Italian patients, the mean genetic distance was 0.099 (ranging from 0.00 to 0.275). The genetic distance between Italian versus non-Italian sequences was 0.099, which was not statistically significant (*p* > 0.05).

### 3.2. Phylodynamic Analysis

Both ML and Bayesian inference approaches indicated that almost all patient sequences clustered with HDV-1 (except for two patients) (Figure 1). Three major clades were inferred, possibly denoting at least two or three major introductions in this part of Italy (Figure 2). These clades were mostly associated with eastern countries, clustering with sequences from Iran, Israel, Russia, and China. Patient 60_ZIYS_Rep_Dem Congo_2020 clustered within an African cluster with sequences from Congo, Cameroon, Nigeria, and Ethiopia. All three major clades appeared to have a common ancestor around the mid-20th century (between 1935 and 1955), which was also supported by the Bayesian skyline analysis, which indicated a sudden increase in the viral population around 1950 (Figure 3a)**.** Using a birth-death model, a clock rate of 3.04 × 10^−4^ substitutions per site per million years was inferred with a 95% Highest Posterior Density (HPD) interval of 3.45 × 10^5^ to 5.72 × 10^−4^.

By implementing a birth-death skyline serial analysis, an effective Reproductive Number (R_e_) was estimated for five distinct time periods (Figure 3). In agreement with our previous analysis, these results indicated a sudden increase from about 1.3 to 4.1 occurring around the 1950s, possibly coinciding with the main introduction of HDV into Italy. The estimated R_e_ value across the different time periods were 1.4, 1.5, 1.4, 1.4, and 4.15, respectively (Figure 3b).

### 3.3. Estimating the Introduction of HDV-1 into Italy

A Bayesian birth-death model with tree calibration based on a sample dating approach indicated multiple original sources of HDV infection (Figure 2). Seven Italian sequences (Pt48, Pt51, Pt54, Pt56, Pt59, Pt65, Pt70) may have originated from a strain dated around 1970. The mean age of these patients was 56 years old (range 42–65). Another group of mainly Italian sequences (Pt4, Pt5, Pt29, Pt43, Pt46) from patients with a mean age of 56 years old (range 31–70) appeared to originate from a strain dated around 1990, while also sharing a common ancestor with Pt76 from the late 1960s. A third group comprising sequences from Italy (*n* = 5; Pt16, Pt39, Pt44, Pt58, Pt66), Albania (*n* = 2; Pt3, Pt69), and Romania (*n* = 1; Pt57) may have been introduced in the late 1950s. The closely related group with five sequences from Romanian (*n* = 3; Pt28, Pt14, Pt68) and Moldavian (*n* = 2; Pt13, Pt26) patients appeared to have a common ancestor from the late 1980s, while also sharing a common ancestor with a Moldavian patient, Pt52, dating from the late 1960s. Similarly, another group with 11 closely related Romanian (*n* = 8; Pt 11, Pt32, Pt33, Pt 47, Pt50, Pt63, P70, Pt78) and Moldavian (*n* = 3; Pt18, P19, Pt71) sequences, including Pt50 and Pt70, who had identical sequences although the respective samples were collected at different times, appeared to have a common ancestor from the late 1970s. These sequences may have also originated from a common ancestor with two Moldavian, one Romanian, and one Russian isolate from a strain that was probably introduced around the late 1950s.

Overall, these results suggest that the Italian HDV sequences analyzed in this study from native Italian and non-Italian patients represent multiple lineages introduced across a wide time period rather than from a common ancestral origin (Figure 1 and Figure 2).

## 4. Discussion

Despite thirty years of horizontal anti-HBV vaccination in Italy, which resulted in a decline in HBsAg carriers from 1.5 to 0.7% of inhabitants [41], the prevalence of HDV/HBV coinfected patients remained stable during the past few decades [29]. Consequently, the HDV screening of HBsAg-positive individuals was recommended, including the monitoring of high-risk groups, such as drug users or incoming infections from regions with high HDV prevalence [42,43]. In our study, HDV sequences from 50 HDV/HBV co-infected patients were analyzed; 20 were from native Italian patients and 30 were from non-Italian patients. All but two sequences were identified as HDV-1. The other two sequences clustered with HDV-5 isolates and belonged to two patients originally from Nigeria and Togo.

Interestingly, no HDV-2 sequences were identified among the evaluated Eastern European patients. This may be due to a low circulation of HDV-2, which is mainly found in the north-eastern part of Russia (Siberia) [44], or to a lower severity of the disease, resulting in a higher probability of recovery than for patients infected with HDV-1. Therefore, the number of patients chronically infected with HDV-2 might be limited.

Le Gal et al. previously reported that HDV-1, the most predominant and diverse of the eight HDV genotypes, can be segregated into four subtypes (1a, 1b, 1c, 1d) [10], while Karimzadeh et al. [11], who performed a phylogenetic analysis using the full-length HDV genome, identified a fifth subtype, HDV-1e. In our phylogenetic analysis, we also included HDV-1e reference strains that had been characterized by analysis of the viral R0 region and full-length genome. Using BLAST, six of our patient HDV isolates were characterized as HDV-1d, two were HDV-1e (Pt67D_GAOV_It_2021 and Pt34_MIDU_Ro_2019), one was HDV-1b (Pt60_ZIYS_Rep_Dem Congo_2020), two isolates from African patients clustered with HDV-5, while the HDV-1 subtypes for the other patient isolates could not be subtyped. However, our phylogenetic analysis identified at least one more sequence as HDV-1e (Pt61D_BAEL_Ro_2020) clustering very closely with Pt34_MIDU_Ro_2019.

The complete classification of HDV-1 is still evolving. Miao et al. [9] inferred that subtypes 1c and 1d were not sufficiently segregated since the internodes in neighbor-joining trees of full-length HDV-1 sequences often displayed bootstrap values of less than 70% [9]. These findings may be related to the high genetic variability of full-length HDV-1. HDV-1d, according to a R0 analysis classification, was the most common subgenotype among our Italian and non-Italian patient isolates, which is in agreement with previous observations in other European countries [10,44,45]. Although the non-Italian isolates exhibited greater genetic variability, the genetic distance between Italian and non-Italian isolates was not statistically significant (*p* > 0.05). Interestingly, some isolates were closely related to those detected 15 to 20 years earlier in Italy (for example: AM902179_dFr2172_It_2006 and AF008413_It_1997). This might suggest that these older isolates were highly transmissible or that our patients were infected during the period when they were circulating.

For our non-Italian patients, the greater genetic variability was probably due to the different origins of the infecting strains. More specifically, non-Italian patients migrated from a range of countries and were, in general, associated with earlier diverging lineages. As demonstrated by Karlsen et al. [44], HDV-1 was introduced into Eastern Europe approximately 180 years ago (95% HPD: 150–210 years) and continues to spread while representing the predominant genotype. Using a Bayesian skyline reconstruction, we observed a relatively recent increase in the viral population (around the 1950s), indicating an ongoing HDV epidemic (Figure 3). Similarly, our birth-death skyline serial analysis exhibited a prominent increase in the reproductive Number (R_e_) from 1.3 to 4.1 in the last 70 years, which is in agreement with observations from another study [44]. This might be the result of greater migration and novel transmission dynamics in a more urban environment, which enable higher transmission rates. Most probably, the circulation of HDV in the 1950s coincided with the circulation of the hepatitis B virus. In fact, there was an increase in blood transfusions during that time, which were not tested for the presence of HBV, and there was also an increase in intravenous drug users [46,47]. However, further studies are required to pinpoint the actual causes of this increase.

For the patients in our study, specific clusters linked to the severity of liver damage were not identified. This may be explained by the fact that the severity of liver damage can be influenced by other factors, such as alcohol use, drug use, and co-infections with other viruses including HIV and high viral load/viraemia. Recently, Romeo and Perbellini reported the relationship between viral load (>600,000 IU/mL) and liver damage [48]. In our study, despite having some patients with viral loads >600,000 IU/mL, similar associations could not be established since the information regarding liver histological findings were not available for all patients.

Of note, two patients from Nigeria and Togo were infected with HDV-5. Even though this genotype was not identified in any other samples, this may represent a source of transmission. Therefore, continued molecular monitoring aimed at evaluating the circulating strains would certainly be worthwhile. Depending on transmission rates, it may also be relevant to include a representation of this genotype when evaluating the effectiveness of antiviral drugs against HDV.

In general, our study focused on the analysis of patients from Regione Lazio. The limited sample size of HDV-positive patients may have impacted our findings and subsequently the conclusion. Moreover, the lack of certain clinical information (date of primary HDV infection, coinfection, superinfection) limited our ability to draw any associations between HDV strains and disease severity or any possible association between HBV genotype/HDV infection and disease severity. A previous study reported that HDV coinfection with the HBV genotype F was associated with a higher level of ALT (alanine aminotransferase), with a greater inflammatory state of the liver [49]. It was also noted that the HDV viral load in patients co-infected with the HBV genotype A was lower than for patients infected with the HBV genotype D. Unfortunately, many patients in our study had an HBV DNA viral load <10 IU/mL due to receiving antiviral therapy, and were therefore not genotyped. Furthermore, our phylogenetic analysis did not include recently identified HDV-1 subtypes (f, g, h, i) [50] as they have not been officially annotated by the international scientific community. These subtypes were mostly identified in patients from Kyrgyzstan, Iran, and Vietnam, countries that are not represented in our patient analysis. Additionally, the genotyping of several of our HDV isolates produced indeterminate results, suggesting that the R0 region is not always adequate for HDV genotyping, due to either the high variability of its genome or due to recombinant forms. It should also be noted that when genotyping our patient HDV sequences, an analysis for recombinant forms was not explored.

Despite the study limitations, our findings on HDV genotyping and its transmission dynamics may aid in the development of prevention and mitigation strategies for HDV-related liver disease in Italy.

## Data Availability

The data presented in this study are available on request from the corresponding author.

## Supplementary Materials

The following supporting information can be downloaded at: https://www.mdpi.com/article/10.3390/v15091791/s1, Figure S1: Maximum Likelihood phylogenetic tree of 50 patient-derived sequences in Italy and 511 reference sequences representing 8 HDV genotypes. Figure S2: Maximum Likelihood tree of HDV sequences with available information on the date of sampling. Table S1: List of HDV Reference sequences. Table S2: Demographic Characteristics of The Study Population.
