# Supplementary material for: Phylogenetic and Phylodynamic Analysis of Delta Strains Circulating in Italy
Source: Viruses. 2023 Aug 23;15(9):1791. doi: 10.3390/v15091791 (PMC10537423; doi:10.3390/v15091791)

Supplementary Figure S1. Maximum Likelihood phylogenetic tree of 50 patient-derived sequences in Italy and 511 reference sequences representing eight HDV genotypes. Italian and non-Italian patient sequences are shown in blue while reference sequences are shown in black. Branch color and width corresponds to 100 bootstrap replicates, based on an HSB color spectrum [RGB $_{min}${204,102,102} | RGB$_{max}${204,102,108}]. The root has been set at midpoint. The tree was inferred with RAxML v8 and visualized with Figtree v1.4.4. Graphic illustration was implemented with Adobe Illustrator 2023.

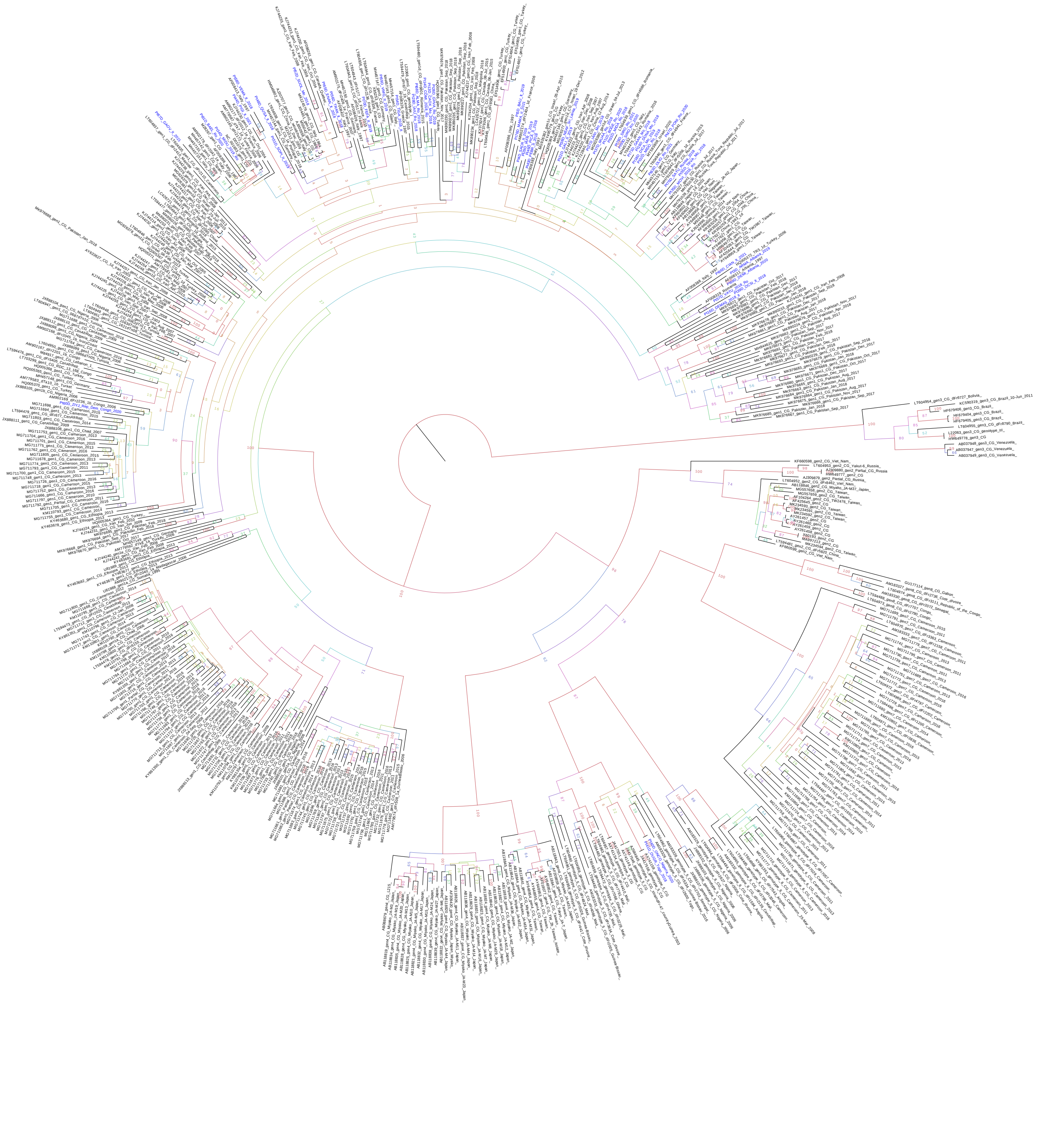

Color Scheme For Single Bootstrap Support Values

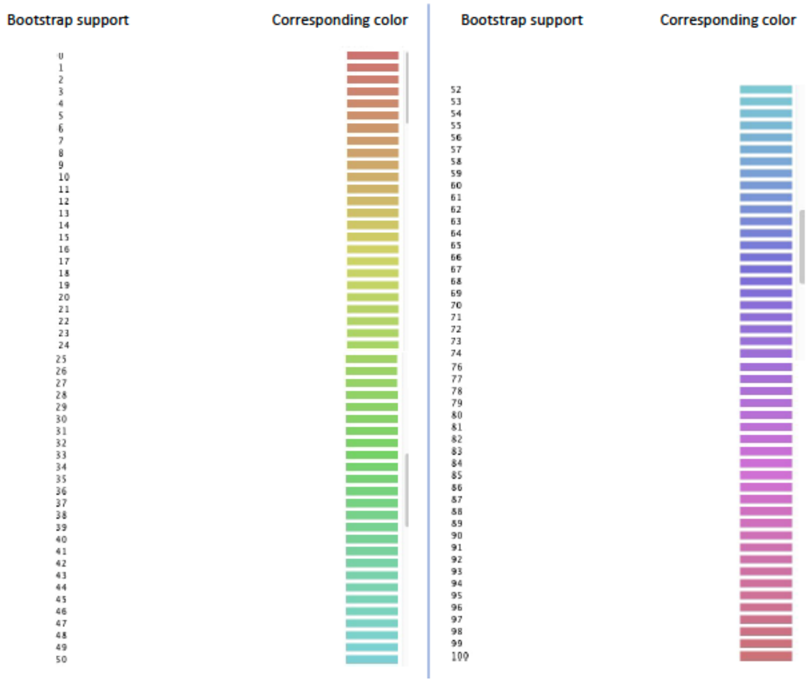

Supplement: Supplementary file 1 [file viruses-15-01791-s001.zip › Supplementary Figure S1.pdf]
