# Supplementary material for: Phylogenetic and Phylodynamic Analysis of Delta Strains Circulating in Italy
Source: Viruses. 2023 Aug 23;15(9):1791. doi: 10.3390/v15091791 (PMC10537423; doi:10.3390/v15091791)

Supplementary Figure S2

For HDV sequences with available information on the date of sampling (n= 102), a Maximum Likelihood tree was generated with 100 bootstrap replicates and partial deletion (90% site coverage cutoff). Reference sequences are colored black, while Italian and non-Italian sequence names are colored blue. The tree was inferred with RAxML v8 and visualized with Figtree v1.4.4. Graphic illustration was implemented with Adobe Illustrator 2023.

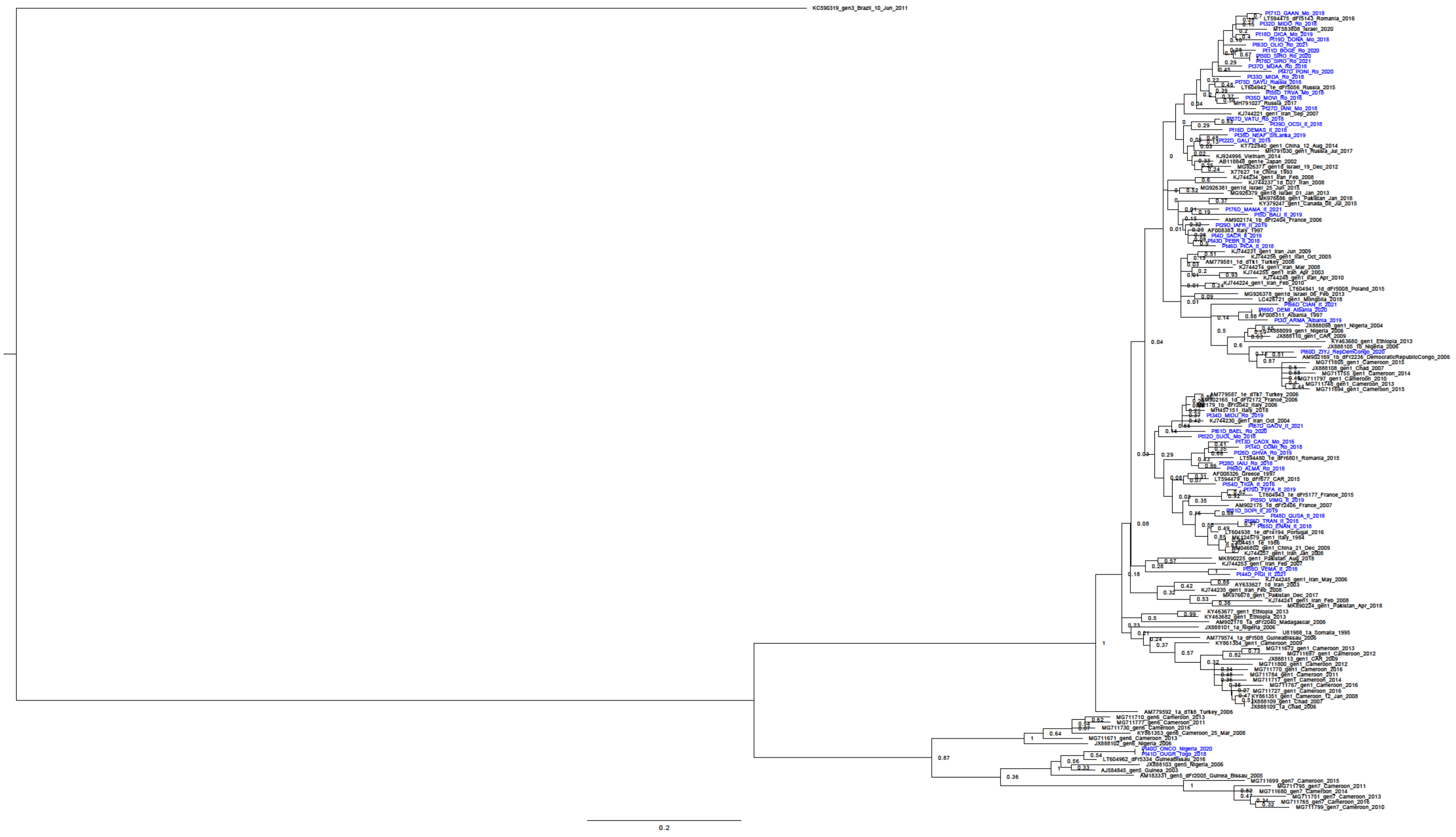

Supplement: Supplementary file 1 [file viruses-15-01791-s001.zip › Supplementary Figure S2.pdf]
