# Supplementary material for: Phylogenetic and Phylodynamic Analysis of Delta Strains Circulating in Italy
Source: Viruses. 2023 Aug 23;15(9):1791. doi: 10.3390/v15091791 (PMC10537423; doi:10.3390/v15091791)
Supplement: Supplementary file 1 [file viruses-15-01791-s001.zip › Supplementary Table S1. List of Reference sequences.pdf]

**List of HDV Reference Sequences**

MK976686_gen1_CG_Pakistan_Jan_2018, MK976678_gen1_CG_Pakistan_Dec_2017, MK976687_gen1_CG_Pakistan_Jan_2018, MK976661_gen1_CG_Pakistan_Aug_2017, MK890233_gen1_CG_Pakistan_Sep_2018, MK976676_gen1_CG_Pakistan_Nov_2017, MK976685_gen1_CG_Pakistan_Jan_2018, MK976667_gen1_CG_Pakistan_Sep_2017, MK976693_gen1_CG_Pakistan_Feb_2018, MK976684_gen1_CG_Pakistan_Jan_2018, MK976675_gen1_CG_Pakistan_Nov_2017, MK976664_gen1_CG_Pakistan_Aug_2017, MK890229_gen1_CG_Pakistan_Sep_2018, MK976680_gen1_CG_Pakistan_Dec_2017, MK976683_gen1_CG_Pakistan_Jan_2018, MK976671_gen1_CG_Pakistan_Oct_2017, MK976669_gen1_CG_Pakistan_Oct_2017, MK976679_gen1_CG_Pakistan_Dec_2017, MK976665_gen1_CG_Pakistan_Aug_2017, MK976663_gen1_CG_Pakistan_Aug_2017, MK976677_gen1_CG_Pakistan_Dec_2017, MK976666_gen1_CG_Pakistan_Sep_2017, MK976682_gen1_CG_Pakistan_Dec_2017, MK976691_gen1_CG_Pakistan_Feb_2018, KJ744241_gen1_CG_Iran_Feb_2008, KY463677_gen1_CG_Ethiopia_2013, MG711800_gen1_CG_Cameroon_2012, KM110795_gen1_CG_Cameroon_, LT594473_gen1_CG_dFr2025_CenAfrRep_, KM110790_gen1_CG_Cameroon_, MG711668_gen1_CG_Cameroon_2014, MG711712_gen1_CG_Cameroon_2013, JX888109_gen1a_CG_Chad_2006, LT594478_gen1_CG_dFr6155_Cameroon, KM110791_gen1_CG_Cameroon, KM110800_gen1_CG_Cameroon, KM110796_gen1_CG_Cameroon, KM110798_gen1_CG_Cameroon, MG711717_gen1_CG_Cameroon_2014, KM110801_gen1_CG_Cameroon_, MG711743_gen1_CG_Cameroon_2013, MG711747_gen1_CG_Cameroon_2013, JX888113_gen1_CG_CenAfrRep_2009, MG711693_gen1_CG_Cameroon_2014, MG711681_gen1_CG_Cameroon_2014, MG711664_gen1_CG_Cameroon_2016, MG711759_gen1_CG_Cameroon_2015, MG711686_gen1_CG_Cameroon_2016, MG711675_gen1_CG_Cameroon_2013, MG711787_gen1_CG_Cameroon_2011, MG711738_gen1_CG_Cameroon_2013, MG711722_gen1_CG_Cameroon_2015, MG711721_gen1_CG_Cameroon_2015, MG711746_gen1_CG_Cameroon_2013, MG711692_gen1_CG_Cameroon_2016, MG711757_gen1_CG_Cameroon_2014, MG711761_gen1_CG_Cameroon_2015, MG711674_gen1_CG_Cameroon_2013, MG711769_gen1_CG_Cameroon_2016, MG711661_gen1_Partial_CG_Cameroon_2016, MG711662_gen1_Partial_CG_Cameroon_2016, MG711756_gen1_CG_Cameroon_2014, MG711744_gen1_CG_Cameroon_2016, MG711745_gen1_CG_Cameroon_2016, MG711665_gen1_CG_Cameroon_2016, MG711663_gen1_CG_Cameroon_2015, AM779574_dFr508_1a_GuineaBissau_2006, MG711732_gen1_CG_Cameroon_2016, MG711676_gen1_CG_Cameroon_2013, MG711778_gen1_CG_Cameroon_2011, MG711785_gen1_CG_Cameroon_2011, MG711802_gen1_CG_Cameroon_2013, MG711696_gen1_CG_Cameroon_2013, JX888100_gen1_CG_Nigeria_2006, JX888101_gen1a_CG_Nigeria_2006, AM902178_dFr2040_1a_Madagascar_2006, MH457149_gen1_CG_Germany, AM779592_dTk8_1a_Turkey_2006, U81988_gen1a_CG_Somalia_1995, KY463682_gen1_CG_Ethiopia_2013, U81989_gen1_CG_Ethiopia_, KY463681_gen1_CG_Ethiopia_2013, KM110792_gen1_CG_Cameroon_, KM110794_gen1_CG_Cameroon_, LT594476_gen1_CG_dFr5405_CenAfrRep_, LT703299_gen1_CG_RDC_13_168_Congo, MG711748_gen1_CG_Cameroon_2013, MG711774_gen1_CG_Cameroon_2013, MG711797_gen1_CG_Cameroon_2010, MG711793_gen1_CG_Cameroon_2011, MG711752_gen1_CG_Cameroon_2013, MG711775_gen1_CG_Cameroon_2013, MG711792_gen1_Partial_CG_Cameroon_2011, MG711678_gen1_CG_Cameroon_2013, MG711805_gen1_CG_Cameroon_2015, MG711753_gen1_CG_Cameroon_2013, MG711666_gen1_CG_Cameroon_2016, MG711762_gen1_CG_Cameroon_2016, MG711726_gen1_CG_Cameroon_2016, MG711705_gen1_CG_Cameroon_2016, MG711718_gen1_CG_Cameroon_2015, MG711704_gen1_CG_Cameroon_2016, MG711701_gen1_CG_Cameroon_2015, AM902169_dFr2236_1b_Congo_2006, MG711694_gen1_CG_Cameroon_2015, MG711803_gen1_CG_Cameroon_2014, MG711755_gen1_CG_Cameroon_2014, MG711700_gen1_CG_Cameroon_2015, AM902167_dFr2201_1b_Congo, MG711698_gen1_CG_Cameroon_2015, MG711764_gen1_CG_Cameroon_2016, AM902168_dFr2045_1b_IvoryCoast, JX888098_gen1_CG_Nigeria_2004, JX888099_gen1_CG_Nigeria_2006, JX888105_gen1b_CG_Nigeria_2006, JX888111_gen1_CG_CenAfrRep_2009, JX888108_gen1_CG_Chad_2007, KM110793_gen1_CG_Cameroon_, LT594479_gen1_CG_dFr677_CenAfrRep_, LT604950_gen1_CG_0866dTn05_Tunisia_, MH457148_gen1_CG_Germany_, HQ005365_gen1_CG_Turkey_, HQ005368_gen1_CG_Turkey_, AM779583_dTk10_1b_Turket, HQ005370_gen1_CG_Turkey_, JX888110_gen1_CG_CenAfrRep_2009,

JX888112_gen1_CG_CenAfrRep_2009, MG711740_gen1_CG_Cameroon_2016, MH457154_gen1_CG_Italy_, MK890235_gen1_CG_Pakistan_Sep_2018, MK890232_gen1_CG_Pakistan_Sep_2018, MK890230_gen1_CG_Pakistan_Sep_2018, MK890228_gen1_CG_Pakistan_Sep_2018, MK890234_gen1_CG_Pakistan_Sep_2018, KJ744237_gen1d_CG_Iran_Feb_2008, MK890227_gen1_CG_Pakistan_Sep_2018, KJ744234_gen1_CG_Iran_Feb_2008, M28267_gen1_CG, MH457142_gen1_CG_Spain_, D01075_gen1_CG, NC_001653_gen1_CG, LT604935_gen1_CG_dFr2110_Cameroon, MH457151_gen1_CG_Italy, AF008398_Italy_1997, AM902165_dFr2172_1d_France_2006, AM779587_dTk7_1e_Turkey_2006, AM902179_dFr2042_1d_Italy_2006, MH457151_Italy_2018, AM902177_dFr2012_1d_France_2006, KJ744230_gen1_CG_Iran_Oct_2004, MH457144_gen1_CG_Germany_, M55042_gen1_CG, MK124579_gen1_CG_Italy_1984, X04451_gen1e_CG_1986, M21012_gen1_CG, AJ307077_gen1_CG_woodchuck5_Italy_, AJ000558_gen1_CG, HM046802_gen1_CG_China_21_Dec_2009, LT604938_gen1_CG_1e_Portugal_2016, AF008407_Italy_1997, LT594480_gen1_CG_dFr6801_Romania_2015, AF008326_Greece_1997, LT594479_dFr677_1b_CenAfrRep_2015, LT604943_gen1_CG_dFr5177_France, AF008409_Italy_1997, LT604943_dFr5177_1e_France_2015, LT604944_gen1_CG_dFr8336_France_, MH457147_gen1_CG_Germany_, X85253_gen1_CG, AM902175_dFr2406_1d_France_2007, MH457143_gen1_CG_Spain_, LT604936_gen1_CG_dFr2136_France_, KJ744220_gen1_CG_Iran_Aug_2007, KJ744221_gen1_CG_Iran_Sep_2007, KJ744222_gen1_CG_Iran_Apr_2008, KJ744223_gen1_CG_Iran_Dec_2008, AB118848_Japan_1e_2002, KJ924996_Vietnam_2014, KR363257_gen1_CG_1048_China_, KR363258_gen1_CG_1054_China_, X77627_gen1_CG, KR363259_gen1_CG_255_China_, X77627_Chine_1993_1e, AF008388_Italy_1997, AF008319_Romania, KF660600_gen1_CG_Viet_Nam_, AB118849_gen1_CG_Nagasaki_JA-N2_Japan_, KF660601_gen1_CG_Viet_Nam_, KF660602_gen1_CG_Viet_Nam_, AF104263_gen1_CG_TW2667_Taiwan_, HW649776_gen1_CG, AF425644_gen1_CG, AY648959_gen1_CG_Taiwan_, AY648958_gen1_CG_Taiwan_, AY648956_gen1_CG_Taiwan_, AY648957_gen1_CG_Taiwan_, AF008383_Italy_1997, AF008386_Italy_1997, AM902174_dFr2404_1b_France_2006, AM779581_dTk1_1d_Turkey_2006, MH791027_gen1_CG_Russia_Jul_2017, MH791029_gen1_CG_Russia_Jul_2017, LT604942_dFr5056_1e_Russia_2015, MH457145_gen1_CG_Germany_, MH457152_gen1_CG_Italy_, LT594475_dFr5143_Romania_2016, MT583808_Israel_2020, LT604940_gen1_CG_dFr4941_France_, HQ005372_TR3_1d_Turkey_2006, AF008311_Albania_1997, MH457153_gen1_CG_Italy_, M92448_gen1_CG_Taiwan_, MG926377_gen1d_CG_Israel_19-Dec_2012, KT722840_gen1_CG_China_12-Aug_2014, LT604937_gen1_CG_dFr2410_Mayotte, AF008413_Italy_1997, KJ744229_gen1_CG_Iran_Dec_2008, KJ744245_gen1_CG_Iran_May_2006, AY633627_CG_1d_Iran_2003, KJ744255_gen1_CG_Iran_Apr_2003, KJ744249_gen1_CG_Iran_Mar_2007, KJ744254_gen1_CG_Iran_Apr_2008, KJ744248_gen1d_CG_Iran_Apr_2010, KJ744247_gen1_CG_Iran_May_2007, KJ744225_gen1_CG_Iran_Sep_2007, KJ744226_gen1_CG_Iran_Sep_2008, KJ744235_gen1_CG_Iran_Feb_2008, KJ744240_gen1e_CG_Iran_Feb_2008, KJ744242_gen1_CG_Iran_Feb_2008, KJ744232_gen1_CG_Iran_Jul_2006, KJ744224_gen1_CG_Iran_Feb_2010, KJ744231_gen1_CG_Iran_Jun_2009, LT604941_gen1_CG__1d_Poland_2015, MG926381_gen1d_CG_Israel_25-Jun_2015, KJ744244_gen1_CG_Iran_Aug_2007, KJ744243_gen1_CG_Iran_Feb_2007, KJ744256_gen1_CG_Iran_Oct_2005, KJ744228_gen1_CG_Iran_Feb_2008, KJ744227_gen1_CG_Iran_Jan_2008, KJ744257_gen1_CG_Iran_Jan_2008, MH457150_gen1_CG_Germany_, L22066_gen1_CG_genotype_I_, KJ744238_gen1_CG_Iran_Feb_2008, MH457146_gen1_CG_Germany_, KY495779_gen1_CG, MG926380_gen1d_CG_Israel_04-Jul_2013, MH791028_gen1_CG_Russia_Tuva_Republic_Jul_2017, MH791030_gen1_CG_Russia_Tuva_Republic_Jul_2017, LT604949_gen1_CG_1063dTn06_Tunisia_, LT604948_gen1_CG_0062dTn11_Tunisia_, MK890226_gen1_CG_Pakistan_Aug_2018, MK890231_gen1_CG_Pakistan_Sep_2018, MG926379_gen1d_CG_Israel_01-Jan_2013, MG926382_gen1c_CG_Israel_05-Apr_2015, HQ005367_gen1_CG_Turkey_, HQ005371_gen1_CG_Turkey_, HQ005369_gen1_CG_Turkey_, HQ005366_gen1_CG_Turkey_, HQ005364_gen1_CG_Turkey_, MK976668_gen1_CG_Pakistan_Sep_2017, MK976694_gen1_CG_Pakistan_Feb_2018, MK976695_gen1_CG_Pakistan_Feb_2018, MK976670_gen1_CG_Pakistan_Oct_2017, KJ744216_gen1_CG_Iran_Jun_2007, KJ744217_gen1_CG_Iran_Jun_2008, KJ744215_gen1_CG_Iran_Jun_2006, LC426721_gen1_CG_Mongolia_2018, LT594474_gen1_CG_dFr2095_China_, MG926378_gen1d_CG_Israel_06-Feb_2013, LT604946_gen1_CG_dTk36_Turkey_, KJ744218_gen1_CG_Iran_May_2006, LT604945_gen1_CG_dFr8581_China_, LT594477_gen1_CG_dFr5985_Georgia_, KJ744253_gen1_CG_Iran_Feb_2007, KJ744214_gen1_CG_Iran_Mar_2008, LT604951_gen1_CG_1621dTn08_Tunisia_, M84917_gen1_CG_Lebanon_1_, KJ744233_gen1_CG_Iran_Feb_2008, KJ744250_gen1_CG_Iran_Oct_2004, LT604939_gen1_CG_dFr4586_Romania_, LC426722_gen1_CG_Mongolia_2018, KY379247_gen1_CG_Canada_08-Jul_2015, KY379246_gen1_CG_Canada_06-Jan_2015, MH844625_gen1_CG_Pakistan_Sep_2017, MK976692_gen1_CG_Pakistan_Feb_2018,

MK976681_gen1_CG_Pakistan_Dec_2017, MK976689_gen1_CG_Pakistan_Jan_2018, MK976690_gen1_CG_Pakistan_Jan_2018, MK976673_gen1_CG_Pakistan_Nov_2017, MK976672_gen1_CG_Pakistan_Oct_2017, KY463680_gen1_CG_Ethiopia_2013, KY463678_gen1_CG_Ethiopia_2013, KM110797_gen1_CG_Cameroon_, KM110799_gen1_CG_Cameroon_, KY861354_gen1_CG_Cameroon_2009, LT604947_gen1_CG_0687dTn10_Tunisia_, JX888104_gen1_CG_Nigeria_2006, MK976674_gen1_CG_Pakistan_Nov_2017, AF098261_gen1_CG_Canada_Quebec_, MK976662_gen1_CG_Pakistan_Aug_2017, MK890224_gen1_CG_Pakistan_Apr_2018, KY463679_gen1_CG_Ethiopia_2013, EF514903_gen1_CG_Turkey_, EF514904_gen1_CG_Turkey_, EF514907_gen1_CG_Turkey_, EF514905_gen1_CG_Turkey_, EF514906_gen1_CG_Turkey_, MK976688_gen1_CG_Pakistan_Jan_2018, MG711767_gen1_CG_Cameroon_2016, MG711703_gen1_CG_Cameroon_2016, MG711716_gen1_CG_Cameroon_2013, MG711770_gen1_CG_Cameroon_2016, MG711766_gen1_CG_Cameroon_2016, KY861355_gen1_CG_Cameroon_2010, MG711683_gen1_CG_Cameroon_2016, MG711684_gen1_CG_Cameroon_2016, MG711736_gen1_CG_Cameroon_2016, MG711728_gen1_CG_Cameroon_2016, MG711798_gen1_CG_Cameroon_2010, MG711749_gen1_CG_Cameroon_2013, MG711794_gen1_CG_Cameroon_2011, MG711796_gen1_Partial_CG_Cameroon_2011, MG711771_gen1_CG_Cameroon_2016, MG711720_gen1_CG_Cameroon_2015, MG711784_gen1_CG_Cameroon_2011, MG711737_gen1_CG_Cameroon_2016, MG711727_gen1_CG_Cameroon_2016, MG711801_gen1_CG_Cameroon_2013, MG711750_gen1_CG_Cameroon_2013, MG711690_gen1_CG_Cameroon_2016, MG711725_gen1_CG_Cameroon_2016, MG711691_gen1_CG_Cameroon_2016, MG711706_gen1_CG_Cameroon_2013, MG711731_gen1_CG_Cameroon_2016, MG711734_gen1_CG_Cameroon_2016, MG711667_gen1_CG_Cameroon_2016, MG711763_gen1_CG_Cameroon_2016, MG711672_gen1_CG_Cameroon_2013, MG711697_gen1_CG_Cameroon_2012, MG711713_gen1_CG_Cameroon_2013, KY861350_gen1_CG_Cameroon_04-Sep_2008, MG711709_gen1_CG_Cameroon_2013, MG711687_gen1_CG_Cameroon_2016, MG711673_gen1_CG_Cameroon_2013, MG711776_gen1_CG_Cameroon_2011, MG711788_gen1_CG_Cameroon_2011, MG711733_gen1_CG_Cameroon_2016, MG711688_gen1_CG_Cameroon_2016, KY861351_gen1_CG_Cameroon_12-Jan_2008, AX741149_genotype_5_CG, JA417546_genotype_5_CG, AJ584845_genotype_5_CG_straindFr-47_countryGuinea_2003, AX741154_genotype_5_CG, JA417551_genotype_5_CG, AM183326_genotype_5_CG_dFr2600_Togo_, LT604962_genotype_5_CG_dFr5334_Guinea-Bissau_2016, AX741159_genotype_5_CG, JA417556_genotype_5_CG, JX888107_genotype_5_CG_Nigeria_2006, JX888103_genotype_5_CG_Nigeria_2006, JX888106_genotype_5_CG_Nigeria_2006, AM183328_genotype_5_CG_dFr2703_Senegal_, LT594482_genotype_5_CG_dFr3634_Cote_dIvoire_, LT604959_genotype_5_CG_dFr4735_Mali_, LT604957_genotype_5_CG_dFr4410_Mali_, LT594483_genotype_5_CG_dFr7068_Ghana_, LT604963_genotype_5_CG_dFr7761_Mali_, AM183331_genotype_5_CG_dFr2005_Guinea-Bissau_, LT594472_genotype_5_CG_dFr3229_Mali_, LT604960_genotype_5_CG_dFr4824_Mali_, LT604956_genotype_5_CG_dFr4066_Mali_, LT604961_genotype_5_CG_dFr5026_Guinea-Bissau_, LT604958_genotype_5_CG_dFr4417_Cote_dIvoire_, LT604967_genotype_6_CG_dFr7057_Cameroon_, LT604968_gen1_CG_dFr7543_Angola_, LT604966_genotype_6_CG_dFr7029_Cameroon_, LT604965_genotype_6_CG_dFr3795_Mali_, LT594485_genotype_6_CG_dFr3046_Cameroon_, AM183329_genotype_6_CG_dFr2627_Nigeria_, LT604964_genotype_6_CG_dFr3006_Cote_dIvoire_, JX888102_genotype_6_CG_Nigeria_2006, LT594484_genotype_6_CG_dFr1594_Angola_, AM183332_genotype_6_CG_dFr2139_CenAfrRep_, MG711671_genotype_6_CG_Cameroon_2013, MG711710_genotype_6_CG_Cameroon_2013, MG711777_genotype_6_CG_Cameroon_2011, MG711730_genotype_6_CG_Cameroon_2016, KY861353_genotype_6_CG_Cameroon_25-Mar_2008, MG711780_genotype_6_CG_Cameroon_2011, AJ309880_gen2_Partial_CG_Russia, HW649777_gen2_CG, LT604953_gen2_CG_Yakut-6_Russia_, AJ309879_gen2_Partial_CG_Russia_, MK234594_gen2_CG_Taiwan_, X60193_gen2_CG, MA997213_gen2_CG, AF104264_gen2_CG_TW2476_Taiwan_, AF425645_gen2_CG, AY261459_gen2_CG, AY261457_gen2_CG, AY261460_gen2_CG, AY261458_gen2_CG, MK234591_gen2_CG_Taiwan_, MK234592_gen2_CG_Taiwan_, MK234593_gen2_CG_Taiwan_, LT594481_gen2_CG_dFr5820_China_, KF660599_gen2_CG_Viet_Nam_, MG557659_gen2_CG_Taiwan_, MG557658_gen2_CG_Taiwan_, AB118846_gen2_CG_Miyako_JA-M37_Japan_, LT604952_gen2_CG_dFr8462_Viet_Nam_, KF660598_gen2_CG_Viet_Nam_, AB118834_gen4_CG_Miyako_JA-

M19_Japan_, AB118835_gen4_CG_Miyako_JA-M20_Japan_, AB118819_gen4_CG_Miyako_JA-M3_Japan_, AB118828_gen4_CG_Miyako_JA-M13_Japan_, AB118821_gen4_CG_Miyako_JA-M5_Japan_, AB118825_gen4_CG_Miyako_JA-M10_Japan_, AB118832_gen4_CG_Miyako_JA-M17_Japan_, AB118838_gen4_CG_Miyako_JA-M24_Japan_, AB118839_gen4_CG_Miyako_JA-M27_Japan_, AB118830_gen4_CG_Miyako_JA-M15_Japan_, AB088679_gen4_CG_L215_, AB118824_gen4_CG_Miyako_JA-M8_Japan_, AB118831_gen4_CG_Miyako_JA-M16_Japan_, AB118823_gen4_CG_Miyako_JA-M7_Japan_, AB118822_gen4_CG_Miyako_JA-M6_Japan_, AF309420_gen4_CG_Miyako_Japan_Miyako_, AB118820_gen4_CG_Miyako_JA-M4_Japan_, AB118833_gen4_CG_Miyako_JA-M18_Japan_, AB118840_gen4_CG_Miyako_JA-M29_Japan_, AB118818_gen4_CG_Miyako_JA-M2_Japan_, AB118827_gen4_CG_Miyako_JA-M12_Japan_, AB118826_gen4_CG_Miyako_JA-M11_Japan_, AB118829_gen4_CG_Miyako_JA-M14_Japan_, AB118836_gen4_CG_Miyako_JA-M44_Japan_, AB118837_gen4_CG_Miyako_JA-M23_Japan_, AB118842_gen4_CG_Miyako_JA-M32_Japan_, AB118843_gen4_CG_Miyako_JA-M33_Japan_, AB118844_gen4_CG_Miyako_JA-M34_Japan_, AB118841_gen4_CG_Miyako_JA-M31_Japan_, AF209859_gen4_CG_Taiwan_, AB118847_gen4_CG_Tokyo_JA-T_Japan_, AY648952_gen4_CG_Taiwan_, AY648953_gen4_CG_Taiwan_, AF018077_gen4_CG_TW_2b_Taiwan_isolate_, AY648954_gen4_CG_Taiwan_, AY648955_gen4_CG_Taiwan_, AB118845_gen4_CG_Miyako_JA-M36_Japan_, MG711789_gen7_CG_Cameroon_2011, MG711680_gen7_CG_Cameroon_2014, MG711760_gen7_CG_Cameroon_2015, MG711765_gen7_CG_Cameroon_2016, MG711685_gen7_CG_Cameroon_2016, MG711711_gen7_CG_Cameroon_2013, MG711714_gen7_CG_Cameroon_2013, MG711772_gen7_CG_Cameroon_2016, MG711751_gen7_CG_Cameroon_2013, MG711773_gen7_CG_Cameroon_2016, MG711739_gen7_CG_Cameroon_2013, MG711790_gen7_CG_Cameroon_2011, MG711741_gen7_CG_Cameroon_2013, MG711779_gen7_CG_Cameroon_2011, MG711735_gen7_CG_Cameroon_2016, MG711781_gen7_CG_Cameroon_2011, MG711804_gen7_CG_Cameroon_2015, MG711729_gen7_CG_Cameroon_2016, MG711754_gen7_CG_Cameroon_2014, MG711768_gen7_CG_Cameroon_2016, MG711723_gen7_CG_Cameroon_2016, MG711679_gen7_CG_Cameroon_2014, MG711786_gen7_CG_Cameroon_2011, MG711689_gen7_CG_Cameroon_2016, MG711783_gen7_CG_Cameroon_2011, MG711799_gen7_CG_Cameroon_2010, MG711724_gen7_CG_Cameroon_2016, MG711758_gen7_CG_Cameroon_2015, MG711791_gen7_CG_Cameroon_2011, MG711699_gen7_CG_Cameroon_2015, AM183333_gen7_CG_dFr2158_Cameroon_, LT604970_gen7_CG_dFr3363_Cameroon_, LT604971_gen7_CG_dFr3836_Cameroon_, MG711695_gen7_CG_Cameroon_2015, KM110803_gen7_CG_Cameroon_, KM110805_gen7_CG_Cameroon_, LT594487_gen7_CG_dFr1650_Cameroon_, KM110804_gen7_CG_Cameroon_, LT594486_gen7_CG_dFr1200_Cameroon_, LT604969_gen7_CG_dFr2202_Cameroon_, MG711682_gen7_CG_Cameroon_2015, LT604972_gen7_CG_dFr4797_Cameroon_, MG711795_gen7_CG_Cameroon_2011, KM110802_gen7_CG_Cameroon_, MG711670_gen7_CG_Cameroon_2013, AM183327_gen8_CG_dFr2736_Cote_dIvoire_, AM183330_gen8_CG_dFr2072_Senegal_, LT604974_gen8_CG_dFr3111_Republic_of_the_Congo_, GU177114_gen8_CG_Gabon_, LT594488_gen8_CG_dFr7707_Congo_, LT604973_gen8_CG_dFr2790_Congo_, L22063_gen3_CG_genotype_III_, HW649778_gen3_CG, HF679404_gen3_CG_Brazil_, HF679405_gen3_CG_Brazil_, HF679406_gen3_CG_Brazil_, KC590319_gen3_CG_Brazil_10-Jun_2011, LT604955_gen3_CG_dFr8790_Brazil_, AB037949_gen3_CG_Venezuela_, AB037947_gen3_CG_Venezuela_, AB037948_gen3_CG_Venezuela_, LT604954_gen3_CG_dFr6727_Bolivia

**List of Dated HDV-1 Reference Sequences**

MK976686_gen1_Pakistan_Jan_2018,MG711748_gen1_Cameroon_2013,MG711797_gen1_Cameroon_2010,MG711755_gen1_Cameroon_2014,MG711694_gen1_Cameroon_2015,MG711805_gen1_Cameroon_2015,JX888108_gen1_Chad_2007,MK890225_gen1_Pakistan_Aug_2018,JX888110_gen1_CAR_2009,JX888099_gen1_Nigeria_2006,KJ744230_gen1_Iran_Oct_2004,MH457151_Italy_2018,AM902179_1e_dFr2042_Italy_2006,MK124579_gen1_Italy_1984,HM046802_gen1_China_21_Dec_2009,KJ744234_gen1_Iran_Feb_2008,KT722840_gen1_China_12_Aug_2014,MG926377_gen1d_Israel_19_Dec_2012,KJ744214_gen1_Iran_Mar_2008,KJ744224_gen1_Iran_Feb_2010,KY379247_gen1_Canada_08_Jul_2015,MG926381_gen1d_Israel_25_Jun_2015,MH791030_gen1_Russia_Jul_2017,KJ744221_gen1_Iran_Sep_2007,LC426721_gen1_Mongolia_2018,KJ744231_gen1_Iran_Jun_2009,KJ744257_gen1_Iran_Jan_2008,KJ744248_gen1_Iran_Apr_2010,KJ744255_gen1_Iran_Apr_2003,KJ744256_gen1_Iran_Oct_2005,MG926378_gen1d_Israel_06_Feb_2013,MG9

26379_gen1d_Israel_01_Jan_2013,KJ744253_gen1_Iran_Feb_2007,JX888098_gen1_Nigeria_2004,KJ744235_gen1_Iran_Feb_2008,KJ744245_gen1_Iran_May_2006,KY463680_gen1_Ethiopia_2013,KY463682_gen1_Ethiopia_2013,MG711800_gen1_Cameroon_2012,JX888109_gen1_Chad_2007,MG711717_gen1_Cameroon_2014,JX888113_gen1_CAR_2009,KY463677_gen1_Ethiopia_2013,MK976678_gen1_Pakistan_Dec_2017,KY861354_gen1_Cameroon_2009,KJ744241_gen1_Iran_Feb_2008,MK890224_gen1_Pakistan_Apr_2018,MG711767_gen1_Cameroon_2016,MG711770_gen1_Cameroon_2016,MG711727_gen1_Cameroon_2016,MG711784_gen1_Cameroon_2011,MG711672_gen1_Cameroon_2013,
MG711697_gen1_Cameroon_2012,KY861351_gen1_Cameroon_12_Jan_2008
