# Supplementary material for: Phylogenetic and Phylodynamic Analysis of Delta Strains Circulating in Italy
Source: Viruses. 2023 Aug 23;15(9):1791. doi: 10.3390/v15091791 (PMC10537423; doi:10.3390/v15091791)
Supplement: Supplementary file 1 [file viruses-15-01791-s001.zip › Supplementary Table S2. Demographic characteristics of study population.pdf]

Table S2. Demographic Characteristics of The Study Population.

Patient	Patient Identifier	Age (y)	Gender	Country	Data collection	HBVDNA (IU/ml)
**Pt3**	**Pt3D_ARMA_2019**	NA	M	AL	18/10/2019	9830
**Pt4**	**Pt4D_SACR_2019**	55	F	It	13/06/2019	<10
**Pt5**	**Pt5D_BALI_2019**	56	F	It	09/04/2019	<10
**Pt11**	**Pt11D_BOGE_2020**	NA	M	Ro	16/10/2020	Not detected
**Pt13**	**Pt13D_CAOX_2016**	44	F	Mo	14/10/2016	Not detected
**Pt14**	**Pt14D_COMI_2018**	59	M	Ro	06/11/2018	<10
**Pt16**	**Pt16D_DEMAS_2018**	58	F	It	10/09/2018	<10
**Pt18**	**Pt18D_DICA_2019**	42	F	Mo	10/07/2019	1910
**Pt19**	**Pt19D_DONA_2018**	56	F	Mo	13/02/2018	<10
**Pt22**	**Pt22D_GALI_2019**	43	F	It	04/12/2019	<10
**Pt26**	**Pt26D_GHVA_2019**	65	M	Ro	02/03/2019	<10
**Pt27**	**Pt27D_IANI_2018**	52	F	Mo	03/11/2018	37
**Pt28**	**Pt28D_IAIU_2018**	34	M	Ro	31/05/2018	<10
**Pt29**	**Pt29D_IAFR_2019**	67	M	It	18/10/2019	56
**Pt32**	**Pt32D_MIDO_2018**	66	F	Ro	06/12/2018	<10
**Pt33**	**Pt33D_MIDA_2018**	NA	M	Ro)	12/09/2018	<10
**Pt34**	**Pt34D_MIDU_2019**	59	M	Ro	30/08/2019	<10
**Pt35**	**Pt35D_MOVI_2016**	40	M	Ro	28/11/2016	<10
**Pt37**	**Pt37D_MUAA_2018**	29	F	Ro	31/07/2018	<10
**Pt38**	**Pt38D_NEAF_2019**	32	M	Sri Lanka	18/11/2019	Not detected
**Pt39**	**Pt39D_OCSI_2018**	47	F	It	02/03/2018	24
**Pt40**	**Pt40D_ONCO_2020**	32	M	Ng	14/01/2020	<10
**Pt41**	**Pt41D_OUGR_2018**	26	M	Tg	24/07/2018	<10
**Pt43**	**Pt43D_PEBR_2018**	70	M	It	13/06/2018	Not detected
**Pt44**	**Pt44D_PIGI_2021**	62	F	It	15/11/2016	<10
**Pt46**	**Pt46D_PICA_2018**	57	M	It	18/12/2018	<10
**Pt47**	**Pt47D_PONI_2020**	51	F	Ro	01/02/2020	<10
**Pt48**	**Pt48D_QUSA_2018**	58	M	It	09/03/2018	<10
**Pt50**	**Pt50D_SIRO_2020**	32	F	Ro	28/09/2020	< 10
**Pt51**	**Pt51D_SOPI_2019**	65	M	It	11/12/2019	<10
**Pt52**	**Pt52D_SUOL_2018**	32	F	Mo	19/12/2018	<10
**Pt54**	**Pt54D_TIGA_2016**	42	M	It	17/10/2016	6937
**Pt55**	**Pt55D_TRVA_2018**	58	F	Mo	12/12/2018	Not detected
**Pt56**	**Pt56D_TRAN_2018**	53	M	It	14/05/2018	<10
**Pt57**	**Pt57D_VATU_2018**	55	M	Ro	17/12/2018	210
**Pt58**	**Pt58D_VEMA_2018**	60	M	It	31/12/2018	<10
**Pt59**	**Pt59D_VIMG_2019**	57	F	It	08/02/2019	45
**Pt60**	**Pt60D_ZIYJ_2020**	59	F	Rep_Dem_Congo	15/02/2020	123
**Pt61**	**Pt61D_BAEL_2020**	65	F	Ro	24/09/2020	<10
**Pt63**	**Pt63D_OLIO_2021**	46	M	Ro	02/04/2021	641
**Pt65**	**Pt65D_ENAN_2018**	52	M	It	30/11/2018	Not detected
**Pt66**	**Pt66D_CIAN_2021**	57	F	It	15/06/2021	Not detected
**Pt67**	**Pt67D_GAOV_2021**	33	M	It	15/06/2021	<10
**Pt68**	**Pt68D_ALMA_2018**	36	M	Ro	10/12/2018	Not detected
**Pt69**	**Pt69D_DEMI_2020**	34	F	Al	13/01/2020	Not detected
**Pt70**	**Pt70D_FEFA_2019**	63	M	It	26/09/2019	Not detected
**Pt71**	**Pt71D_GAAN_2018**	40	M	Mo	01/03/2018	<10
**Pt75**	**Pt75D_SAYU_2018**	42	F	Ru	21/08/2018	80620
**Pt76**	**Pt76D_MAMA_2021**	65	M	It	26/05/2021	Not detected
**Pt78**	**Pt78D_SIRO_2021**	33	F	Ro	24/04/2021	20

Ro, Romania; Al, Albania; NG, Nigeria; It, Italy; Tg, Togo; Rep_Dem_Congo, Democratic Republic of the Congo; Mo, Republic of Moldova; Ru, Russian Federation.
